# Pain assessment on a numerical scale with uncertainty intervals: a proof-of-concept simulation study

**DOI:** 10.3389/fpain.2025.1555185

**Published:** 2025-05-30

**Authors:** Markus Huber, Ulrike Stamer

**Affiliations:** ^1^ Department of Anaesthesiology and Pain Medicine, Inselspital, Bern University Hospital, University of Bern, Bern, Switzerland; ^2^ Department of BioMedical Research, University of Bern, Bern, Switzerland

**Keywords:** pain assessment, pain intensity, numerical rating scale, visual analog scale, simulation study

## Abstract

**Background:**

Reliable and validated scores assessing pain-related outcomes are an essential component of pain management. Point estimates, e.g., on the numeric rating scale (NRS), are widely used. Given the broad spectrum of physiological and psychological factors involved in a patient's pain experience, these point estimates entail inherent uncertainty. To account for this uncertainty, we propose a statistical framework featuring uncertainty intervals on a numerical scale assessing pain intensity.

**Methods:**

We describe a non-parametric statistical method to estimate the effectiveness of a pain intervention when patients provide an uncertainty interval of pain intensity rather than a single point estimate. We consider pain intensities on a generic numerical pain scale (NPS) ranging from 0 to 10 and illustrate the method's performance with proof-of-concept simulation studies and sensitivity analyses.

**Results:**

The simulation studies demonstrate that the non-parametric method can derive correct estimates of the average treatment effects in idealized settings. Importantly, the method can represent the traditional pain assessment with point estimates when the widths of the uncertainty intervals are gradually decreased toward the mean of the uncertainty interval.

**Conclusion:**

We proposed a new statistical framework to account for patient-specific uncertainties in pain intensity as measured on a numerical scale. The clinical importance of the method lies in its ability to reflect the large heterogeneity of individual pain experiences and the possibility of investigating pain-related aspects that go beyond a traditional pain assessment with point estimates. Future clinical studies are required to assess the method's clinical validity and utility.

## Introduction

The reliable assessment of a patient's pain by validated instruments constitutes a cornerstone of modern pain management (1). Several assessment tools are available, e.g., the numeric rating scale (NRS) or the visual analog scale (VAS). However, it is sometimes difficult for patients to decide on a specific NRS measure. Pain intensity is often dynamic and with more or less pronounced fluctuations over time. These unidimensional scales provide the main components for validated multidimensional questionnaires such as the Brief Pain Inventory (BPI) or the PAIN OUT questionnaire developed for patients either suffering from chronic pain or acute postoperative pain (2, 3).

In this brief research report, we study a specific statistical aspect of current pain measurement tools, notably the NRS and the VAS. In particular, we consider the fact that current pain assessment tools traditionally quantify the pain intensity by a point estimate—be it for a single question or as part of multiple point estimates of a questionnaire. Given the multidimensional aspects and multifactorial processes involved in an individual's pain experience, we examine the possibility of extending the point estimate-based pain assessment using a patient-specific uncertainty interval of pain intensity. This extension is motivated by clinically relevant aspects of pain assessment that go beyond the current abilities of pain assessments with point-based estimates: For example, are the patients more or less certain of their experienced pain intensities after an intervention? Uncertainty intervals on a pain scale of interest could provide additional, clinically relevant insights into the patients’ pain perception that would not require additional items on a questionnaire or additional dichotomous items.

Therefore, we aim to propose a statistical method that allows the incorporation of individual-level uncertainty in pain intensity as expressed in intervals on a numerical pain scale (NPS). Using a suite of simulation studies, we provide a step-by-step introduction to the method and its performance. We further compare the accuracy of the method to traditional pain assessments with point-based estimates. We conclude by discussing clinically relevant aspects when implementing the method in a real-world setting and the need to examine the method's clinical utility and validity in future studies.

## Methods

### Objective

The objective of the statistical method is to provide a mean estimate and an associated 95% confidence interval (CI) of the average treatment effect (ATE) in a before-and-after setting where a cohort receives a pain-related treatment. Importantly, individual pain intensities are expressed with uncertainty intervals on a numeric pain scale (NPS) of arbitrary units at the two time points. To mimic the established pain scores, the NPS ranges from 0 to 10 in this study.

### Illustration example

**Figure 1:**
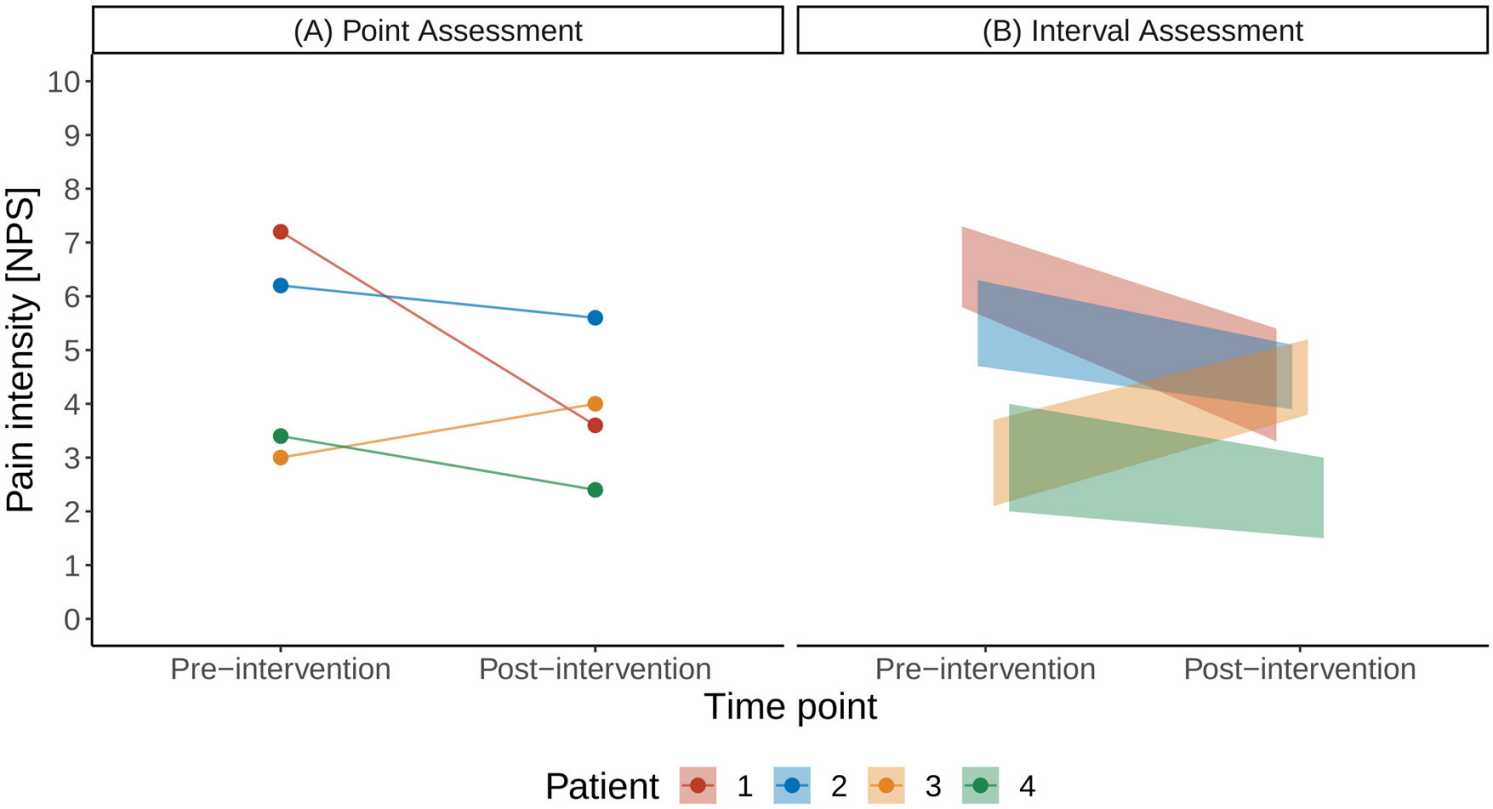
Illustration of a traditional pain assessment with a single point estimate **(A)** and with the interval assessment **(B)** on a numerical pain scale (NPS).

As a motivational example for the subsequent technical description, we illustrate the case of a point estimate using a small illustration example shown in Figure 1: Four patients reported their pain intensity on the numerical pain scale before and after an analgesic intervention (Figure 1A). The scenario of interest here is the case when patients can provide uncertainty intervals on the pain scale, instead of point estimates (Figure 1B).

### Statistical framework

**Figure 2:**
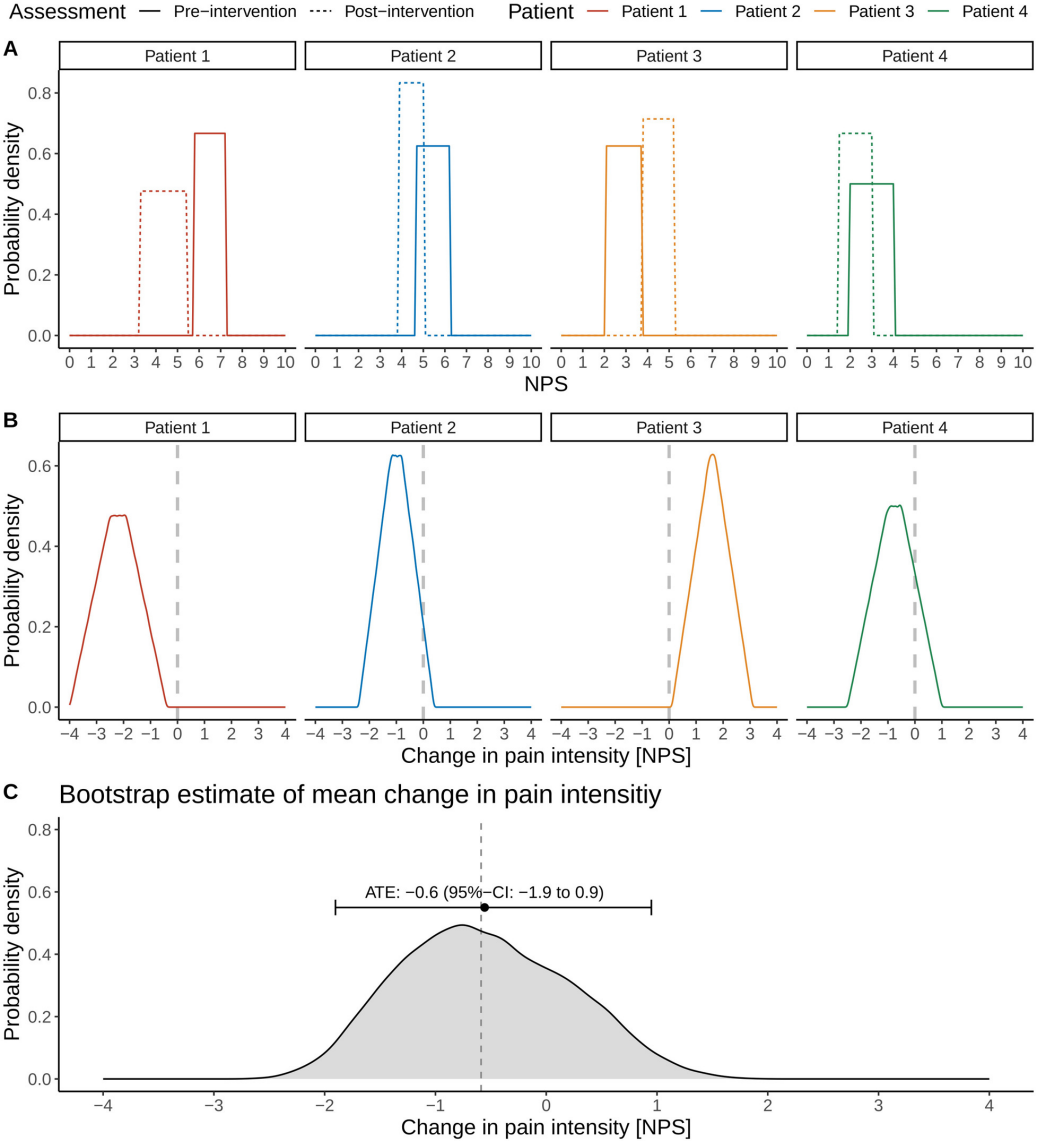
Illustration of the different steps involved in estimating the average treatment effect (ATE) and associated 95% confidence interval (CI) in pain intensities in the presence of uncertainty intervals on a numerical pain scale (NPS). Distributions of pre- and post-intervention pain intensity levels **(A)** and the associated distributions of the changes in pain intensities for individual patients **(B**, **C)**. Bootstrap estimation of the ATE and its 95% CI. The vertical dashed line refers to the true ATE of the simulated cohort.

We introduce the different steps involved in the proposed framework in Figure 2 using the data of the small cohort shown in Figure 1. Figure 2A illustrates the uniform probability distribution of pre- and post-intervention pain intensities. The pre- and post-intervention pain intensities are considered uniform random variables where the lower and upper boundaries are defined by the patients’ pain intensity intervals. For example, Patient 1 stated his pre-intervention pain levels between 5.8 and 7.3 units on the NPS and his post-intervention pain levels between 3.3 and 5.4 units.

We can now consider the patient-specific treatment effects. In the case of traditional point estimates, these would correspond to a single number: For example, the treatment effect for Patient 1 would be a pain reduction of 3.6 units on the NPS scale (pre-intervention pain level of 7.2 units and post-intervention level of 3.6 units; Figure 1A). In the case of uncertainty intervals, the patient-specific treatment effects are random variables, whose distributions are shown in Figure 2B. The triangular shape of the resulting distributions results from the so-called convolution of the patient-specific pre- and post-intervention pain intervals.

The convolution is best explained for the case where the uncertainty intervals of pre- and post-intervention pain intensities do not overlap, such as in the case for Patient 3; this patient stated pre-intervention pain levels between 2.1 and 3.7 units and post-intervention pain levels between 3.8 and 5.2 units. Note that Patient 3 showed an overall increase in pain levels after the intervention. The triangular shape of Patient 3's distribution of the treatment effect can be derived by first considering the smallest post-intervention pain level (3.8 units) and the largest pre-intervention pain level (3.7 units), resulting in a small increase in pain level of 0.1 units, corresponding to the lower left orange corner of the triangle in Figure 2B. Conversely, considering the largest post-intervention pain level (5.2 units) and the smallest pre-intervention pain level (2.1 units) results in the largest possible change in pain intensity of 3.1 units, which corresponds to the lower right orange corner of the triangle in Figure 2B.

However, these changes in pain intensities are very unlikely and simply denote the possible range of patient-specific treatment effects, as these correspond to the lower and upper boundaries of the pain uncertainty intervals. Other values of the treatment effect are much more likely as different values of the treatment effect can result from subtracting a particular post-intervention pain level from a particular pre-intervention pain level: If one computes all possible differences of post- and pre-intervention pain levels for Patient 3, the resulting orange shape is the orange triangular in Figure 2B.

### Statistical estimation

We infer the ATE's sampling distribution using bootstrap sampling in a three-stage process illustrated in the Supplementary Material:

• First and related to *sampling uncertainty*, we sample four patients from the original cohort with replacement.
• Second and related to *patient-specific uncertainty* in pain intensities as expressed in the pain uncertainty intervals, we draw for each patient one sample from its (uniform) post-intervention distribution and one sample from the (uniform) pre-intervention distribution and calculate the individual treatment effect. The individual treatment effect corresponds to one sample of the probability distributions shown in Figure 2B.
• Third, we calculate the ATE of the bootstrapped cohort by averaging the individual treatment effects.

We repeat the ATE calculations for a given bootstrapped cohort 100 times (Step 2 above). We then repeat the bootstrap sampling of the cohort (Step 1 above) also 100 times, resulting in an empirical, non-parametric sampling distribution of the ATE from which we can calculate the ATE of −0.6 (95% CI: −2.0 to 1.0) units as illustrated in Figure 2C.

All simulations were computed in R version 4.2.3 (4).

### Proof-of-concept simulation

**Figure 3:**
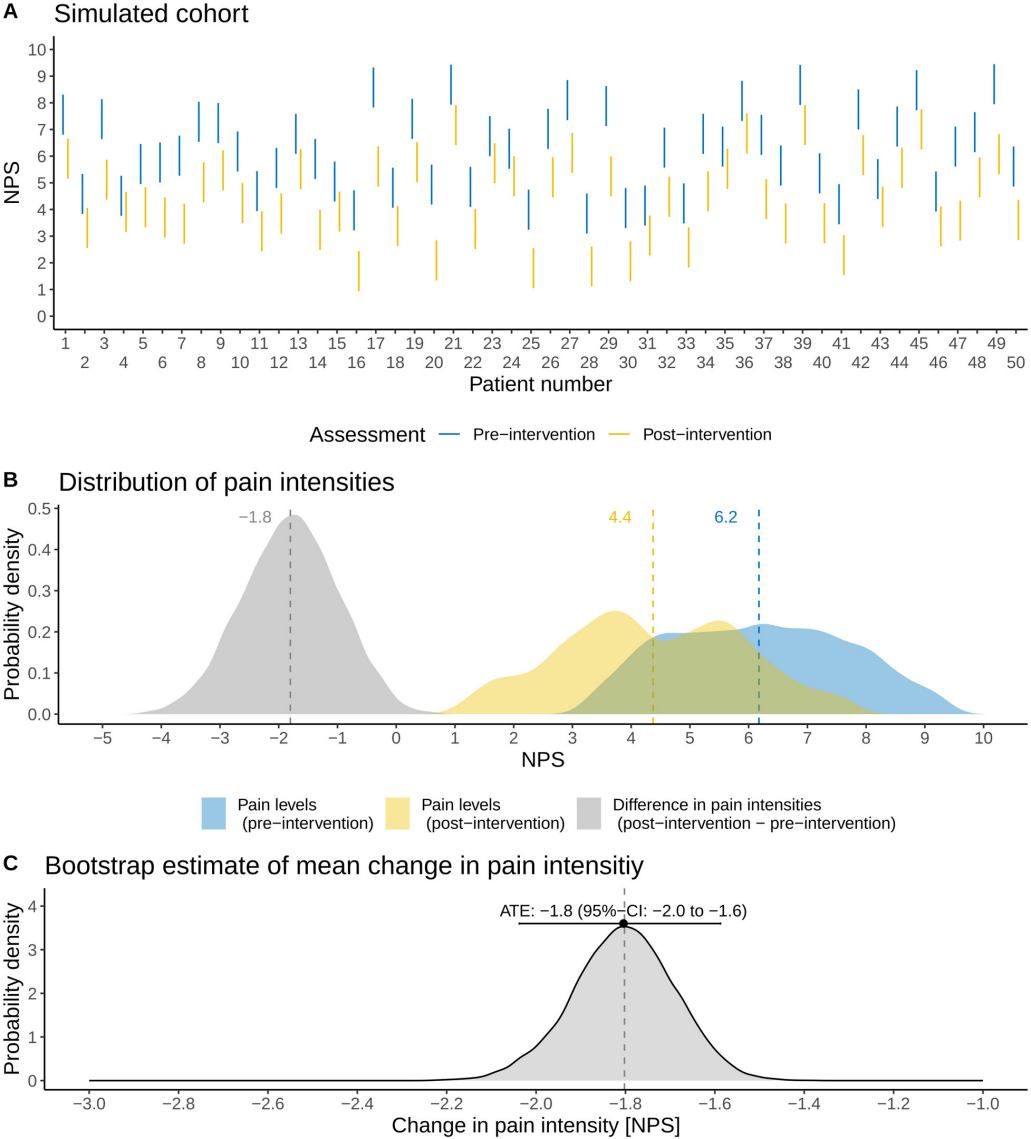
Application of the methodology to an idealized, simulated cohort (*N* = 50 patients). **(A)** Illustration of the patients’ assessed pain intensity intervals before and after a pain-related intervention on a numerical pain scale (NPS). **(B)** Probability distributions of pre- and post-intervention pain intensities as well as the associated changes in pain intensities (post-intervention minus pre-intervention). **(C)** Non-parametric sampling distribution of the average treatment effect (ATE) derived with the method outlined in this brief research report. The mean and 95% CI of the ATE are shown, and the vertical dashed line refers to the true ATE of the simulated cohort.

As proof of concept, we demonstrate the application of the methodology for an idealized, simulated cohort of 50 patients. For simplicity and illustration purposes, the uncertainty intervals are equal for each patient and are chosen as 3 units on the NPS. The true population ATE was fixed to −2 units (a pain reduction). The sample size was chosen such that the uncertainty intervals of individual patients could be comprehensively illustrated (Figure 3)—featuring both overlapping and non-overlapping uncertainty intervals—as well as having enough power to detect the ATE. No formal sample size calculation was performed, as the illustration of the uncertainty intervals was the primary objective of the proof-of-concept simulation rather than statistical efficiency.

Figure 3A shows the distribution of pain intensities (*N* = 50). The average pain intensities in the simulated cohort are 6.2 (standard deviation: 1.5) units for pre-intervention and 4.4 (standard deviation: 1.5) units for post-intervention (Figure 3A). The ATE of the simulated cohort is a pain reduction of −1.8 units. Note that there is a difference of 0.2 units from the true, underlying population ATE: this difference results simply from sampling. Applying the non-parametric method outlined above, we estimate the ATE as −1.8 (95% CI: −2.0 to −1.6), thus correctly estimating the ATE of the simulated cohort (Figure 3C).

### Sensitivity analyses

**Figure 4:**
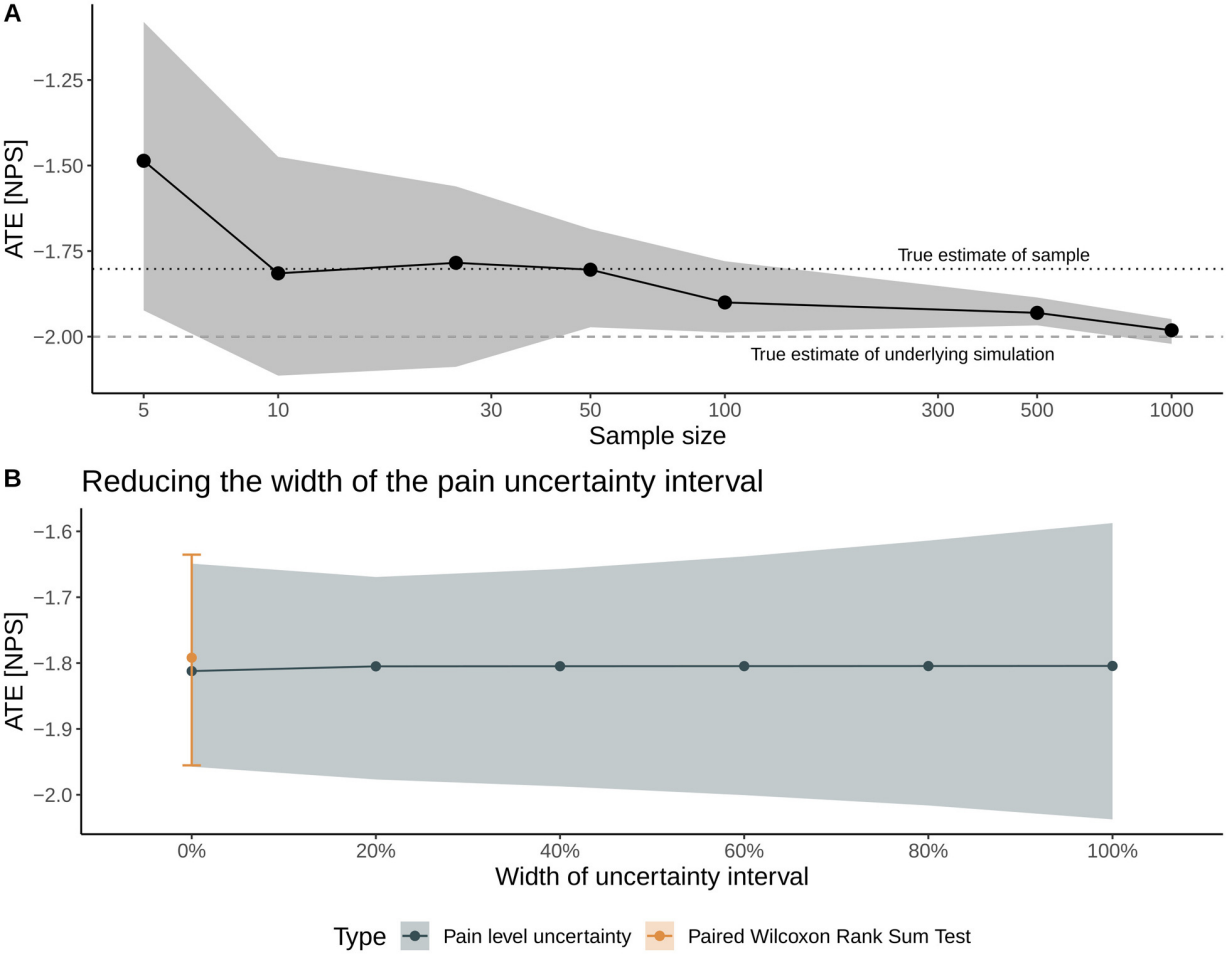
Sensitivity analyses to examine the method's performance with respect to sample size **(A)** and to the width of the uncertainty interval on a numerical scale for pain intensity **(B)**. In Panel **(A)**, solid dots represent the mean average treatment effect (ATE), and the gray shading represents the inferred 95% CI. The dotted and dashed lines denote the true ATE of the sample and the population, respectively. In Panel **(B)**, the width is gradually decreased from the full width (denoted by a 100% width) toward the mean value of the uncertainty interval (denoted as 0% width), allowing for benchmarking the method with a traditional statistical test for nominal, paired data (Wilcoxon rank sum test).

We further investigate the method's performance in two sensitivity analyses shown in Figure 4.

The first sensitivity analysis considers the ATE's estimate and 95% CI with respect to sample size, where we repeat the above proof-of-concept simulation with sample sizes ranging from *N* = 5 patients up to *N* = 1,000 patients; all other parameters of the simulations—notably the widths of the uncertainty intervals—are the same. With respect to the cohort's sample size, the mean estimate of the ATE is sensitive to sampling uncertainty for sample sizes below 100 patients but correctly estimates the ATE of the particular sample (i.e., −1.8 units). Increasing the sample size results in a correct estimate of the population ATE of −2 units (Figure 4A).

The second sensitivity analysis examines the impact of the width of the uncertainty interval on the treatment effect estimates: We incrementally shrink the width of the uncertainty interval (a 100% width corresponds to the patient-reported interval width) toward the interval's mean value (corresponding to 0% width). Thus, this sensitivity aims to mimic the convergence toward a traditional point measure of pain intensity. At 0% width, the results are compared with and benchmarked against the estimate from a paired Wilcoxon rank sum test. Figure 4B illustrates that the uncertainty of the inferred treatment effect decreases for smaller interval widths and highlights that the estimates derived with the non-parametric method outlined here agree very well with the median and 95% CI of the ATE derived with the paired Wilcoxon rank sum test.

## Discussion

Point estimates of pain intensity with a single value on a numerical scale, e.g., the NRS or VAS, play a pivotal part in modern pain assessment. However, multiple factors render this task of objectifying the subjective pain experience a challenging task (5). For example, these factors can relate to cultural, social, and environmental factors interacting with the nature of the pain experienced by a patient (6). There are multiple ways in practice to quantify the associated uncertainty in these point estimates, for example, by providing a more detailed context to the pain intensity of interest, e.g., by providing a specific recall period (current pain, pain intensity in the last 24 h, or minimum and worst pain experienced with the last 24 h) (7, 8).

In this brief research report, we proposed a statistical method that incorporates patient-specific uncertainties related to the broad spectrum of physiological and psychological factors involved in pain directly for the question of interest. The method enables patients to report their pain intensities with uncertainty intervals rather than a single value. As this is the first attempt to incorporate uncertainty intervals in estimates of pain intensities, we first focused on proof-of-concept simulations with associated sensitivity analyses to introduce the method. To the best of our knowledge, there are no real-world observational or interventional studies collecting such data. Overall, Figures 3 and 4 demonstrate the method's validity in estimating the ATE and associated uncertainties in the idealized, proof-of-concept simulations.

We emphasize that this proof-of-concept simulation study does not discuss the clinical validity and clinical utility of the proposed method. These topics require additional future work as the main objective of this brief research report is to introduce and discuss the statistical possibility of working with uncertainty intervals in pain intensities. When moving from this proof-of-concept study toward clinical implementation, special attention will be required in the explanation of the exact meaning of the uncertainty intervals to the patients to ensure a consistent and transparent implementation. For example, when asking patients for the uncertainty intervals regarding their pain level, one could imagine explaining that the uncertainty levels relate to their confidence in a particular pain item, for example, in current pain. Concretely, one could ask: “On this scale ranging from 0 (no pain) to 10 (worst possible pain), what is the level of your current pain? You may indicate your certainty by either providing a single number as an answer (very certain) or a range of pain levels that you feel representative of your current pain.” Another use case of the proposed methodology could be to quantify the patient's perception of uncertainty regarding a particular pain item, for instance worst pain experienced over the last 24 h. We note, however, that the exact definition of the uncertainty intervals in a clinical setting requires further discussion with clinicians.

Importantly, this study does not question the current practice of assessing pain intensities with point estimates. It rather demonstrates potential future avenues for pain assessment and possibilities to extend the current practice of point estimates by incorporating patient-specific uncertainty intervals. There are several advantages of these uncertainty intervals: First, the ranges can assess more individualized pain experiences and may differ before and after an intervention. Additionally, the uncertainty intervals can be contrasted to a minimum clinically significant difference in pain intensity in a straightforward fashion (9). Second, the proposed method offers the possibility to infer ranges of pain intensities—for example, minimum and maximum pain—using a single question rather than multiple questions. Thus, the methods allow for incorporating additional information without adding more items related to the uncertainty of pain experience (e.g., binary or ordinal items where uncertainty can be explicitly stated). The method therefore avoids the issue of multiple comparisons and alpha-inflation when multiple hypotheses are tested.

Third and in the context of the general difficulty of analyzing pain scores (10), the proposed non-parametric method makes only the assumption that the uncertainty intervals imply uniformly distributed pain intensities—the ATE estimation and its associated uncertainty can also be performed with different assumptions about the nature of the sampling distribution. Fourth, the method can be easily adopted to the discrete NRS by replacing the uniform distribution of the uncertainty intervals with discrete probability mass functions. In terms of practical implementation in a questionnaire, the uncertainty intervals could be collected on either the NRS or VAS scale—the proposed method works for both discrete ordinal scales and continuous scales.

This analysis features some inherent limitations. Notably, a detailed investigation of all possible statistical aspects of the framework was beyond the scope of this study. In particular, the robustness and consistency of the estimator need further analysis with theoretical underpinnings. Additionally, this brief research report primarily focuses only on the statistical aspects of pain assessment and could not discuss the context of pain definitions and classifications as outlined in the International Statistical Classification of Diseases and Related Health Problems (ICD-11).

To conclude, we illustrated a proof-of-concept simulation study of a statistical framework extending the current practice of assessing pain intensity with point estimates to incorporate inherent individual uncertainties using uncertainty intervals on a numerical pain scale.

## Data Availability

The original contributions presented in the study are included in the article/Supplementary Material; further inquiries can be directed to the corresponding author.
