# Supplementary material for: Pain assessment on a numerical scale with uncertainty intervals: a proof-of-concept simulation study
Source: Front Pain Res (Lausanne). 2025 May 30;6:1555185. doi: 10.3389/fpain.2025.1555185 (PMC12163017; doi:10.3389/fpain.2025.1555185)
Supplement: Supplementary file 1 [file Datasheet1.docx]

Supplementary Material to:

Pain assessment on a numerical scale with uncertainty intervals:

**A proof of concept simulation study**

Markus Huber^1^, Ulrike Stamer^1,2^

^1^ Department of Anaesthesiology and Pain Medicine , Inselspital, Bern University Hospital, University of Bern, Freiburgstrasse, 3010 Bern, Switzerland.

^2^ Department of BioMedical Research, University of Bern, Freiburgstrasse, Murtenstrasse 28, 3008 Bern, Switzerland.

*** Correspondence:**Markus Huber, Dr. sc. ETH, Department of Anaesthesiology and Pain Medicine, Inselspital, Bern University Hospital, University of Bern, Bern, Switzerland, Freiburgstrasse 10, 3010 Bern, Switzerland, Tel: +41 31 664 12 15, Email: [markus.huber@insel.ch](mailto:markus.huber@insel.ch)

**Keywords**: pain assessment, pain intensity, numerical rating scale, visual analogue scale, simulation study

**
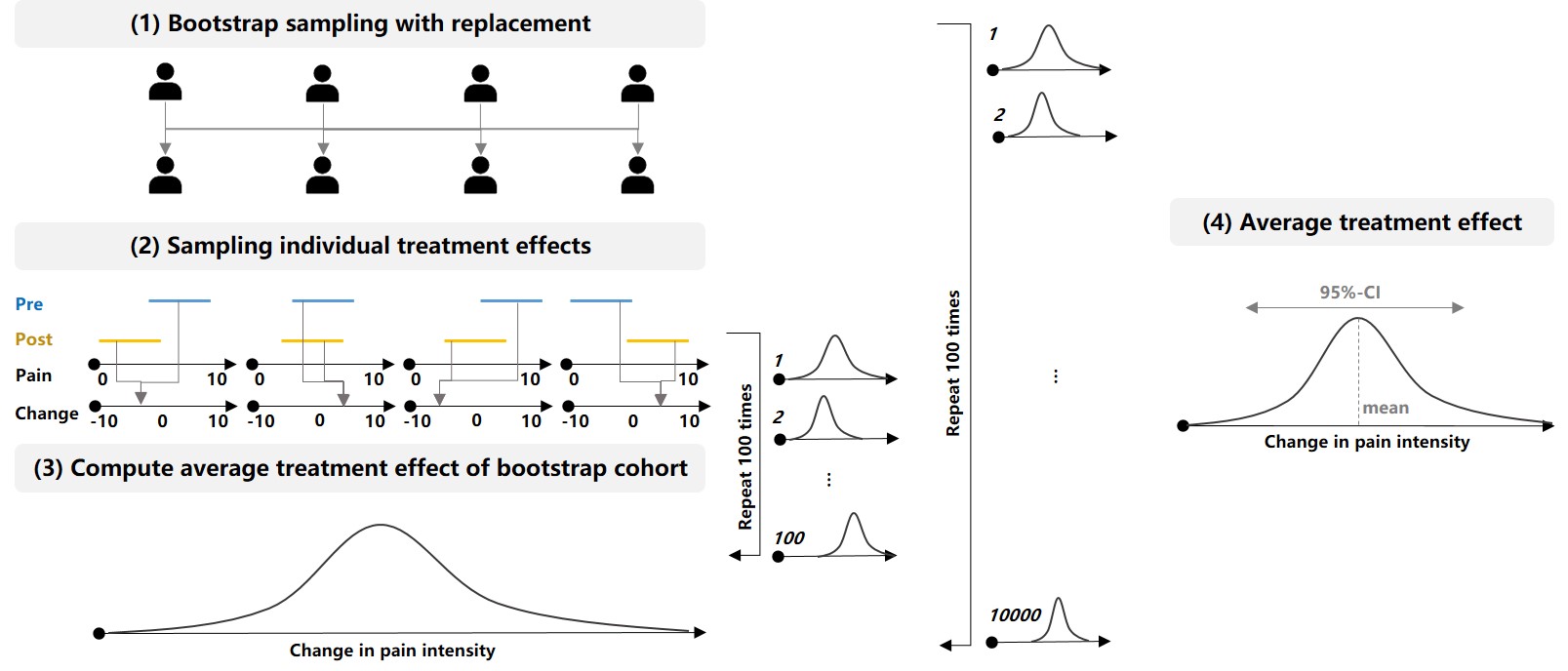
**

**Supplementary Figure.** Illustration of the non-parametric bootstrap estimation of the average treatment effect and associated 95%-confidence intervals in the presence of patient-specific uncertainty intervals.
